# Accumulated genetic mutations leading to accelerated initiation and progression of colorectal cancer in a patient with Gardner syndrome: A case report

**DOI:** 10.1097/MD.0000000000025247

**Published:** 2021-04-02

**Authors:** Xiaoqiang Gu, Xin Li, Jiahua Xu, Jinzu Yang, Hongwei Li, Qing Wu, Jianxin Qian

**Affiliations:** Department of Oncology, Longhua Hospital Affiliated to Shanghai University of Traditional Chinese Medicine (TCM), Shanghai, China.

**Keywords:** colorectal carcinoma, Gardner syndrome, genetic mutations

## Abstract

**Rationale:**

Gardner syndrome is a rare autosomal dominant disorder with a high degree of penetrance, which is characterized by intestinal polyposis, osteomas, and dental abnormalities. Majority of patients with Gardner syndrome will develop colorectal cancer by the age of 40 to 50 years. Mutations in the adenomatous polyposis coli gene are supposed to be responsible for the initiation of Gardner syndrome.

**Patient concerns:**

A 22-year-old Chinese female was admitted to our hospital due to abdominal pain and bloody stool.

**Diagnosis:**

The patient presented with multiple intestinal polyposis, desmoid tumors, and dental abnormalities was diagnosed as Gardner syndrome and further examination revealed a colon tumor.

**Interventions and outcomes:**

Patients were implanted with stents to alleviate bowel obstruction, and were treated with oxaliplatin combined with 5-Fu for 4 cycles, but the efficacy was not good. We performed next generation sequencing of 390 genes for the tumor specimens. We detected adenomatous polyposis coli E1538Ifs∗5, KRAS G12D, NF1 R652C, loss of SMAD4, TP53 R175H, IRF2 p.R82S, TCF7L2 p.A418Tfs∗14, and SMAD4 p.L43F in this patient.

**Lessons:**

We reported serial mutations in key genes responsible for initiation and progression of colorectal cancer from a patient with Gardner syndrome.

## 1 Introduction

Gardner syndrome (GS) is a rare autosomal dominant disorder with a high degree of penetrance, which was first described by Devic and Bussy in 1912 and Eldon J Gardner in 1951.^[1]^ GS is characterized by the presence of multiple intestinal polyposis, extra-colonic tumors (including osteomas, thyroid cancer, epidermoid cysts, and desmoid tumors), and dental abnormalities.^[2,3]^ GS is also known as a familial adenomatous polyposis (FAP) subtype.^[4]^ FAP is an inherited condition in which numerous adenomatous polyps arising mainly in the epithelium of the large intestine.^[5,6]^ The root cause of FAP is supposed to be a genetic mutation – adenomatous polyposis coli gene (*APC*) defects on chromosome 5q21.^[7]^ Increasing numbers of mutations of the *APC* gene have been identified in families with FAP, as well as in individuals with GS.^[8]^ In addition to *APC* gene defects, next generation sequencing (NGS) is able to discover more potential oncogenic drivers and clinical therapeutic targets.^[9]^

## 2 Case presentation

**Figure 1:**
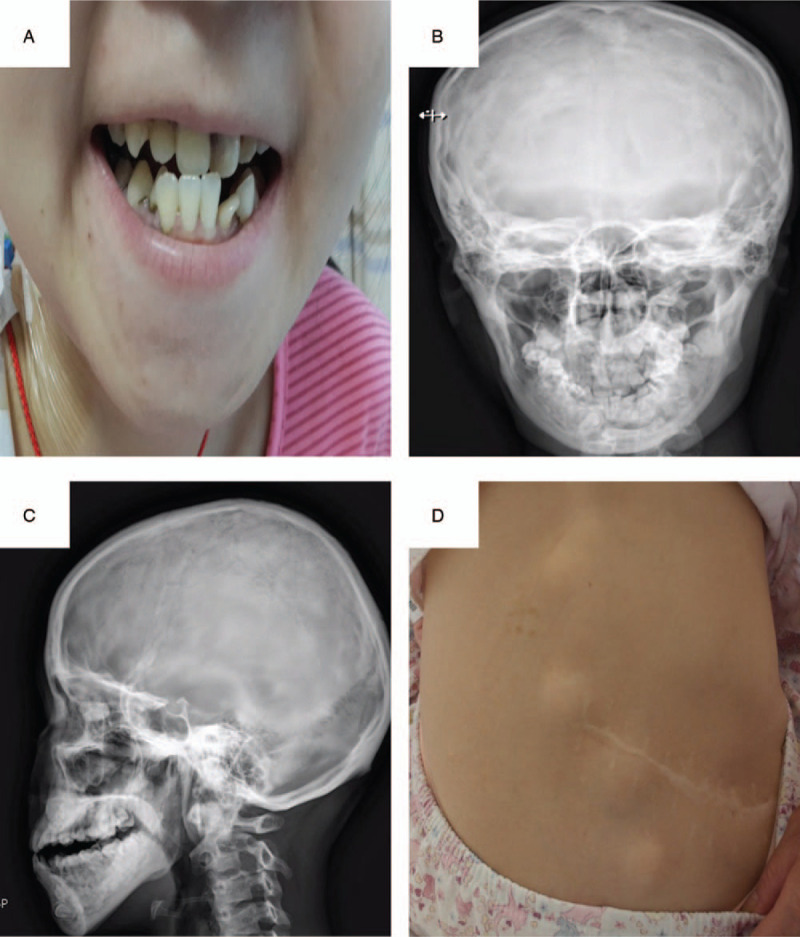
Dental abnormality of a patient with GS. (A) Initial intraoral clinical aspect of the patient. (B and C) Panoramic radiograph showing the presence of supernumerary teeth. (D) Photograph showing epidermoid cyst over the lower back.

**Figure 2:**
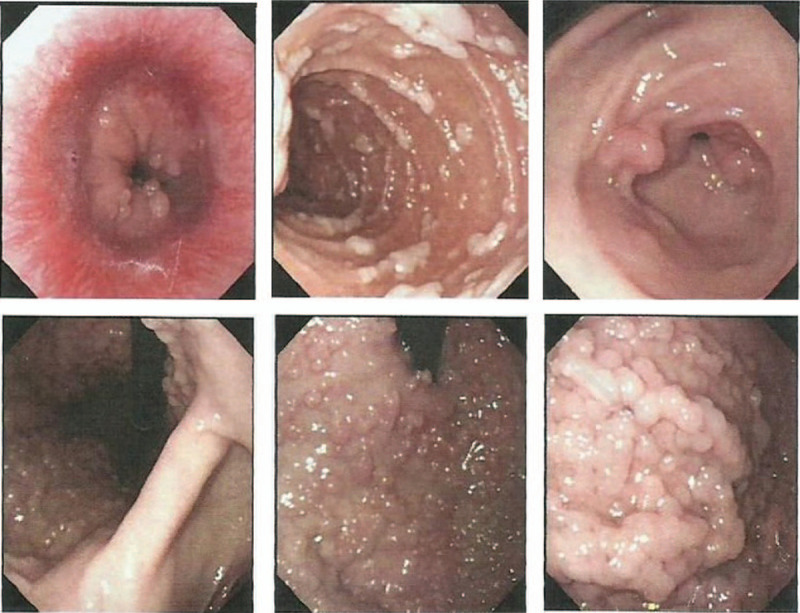
Colonoscopy identifying a malignant appearing lesion and leading to construction of the bowel.

**Figure 3:**
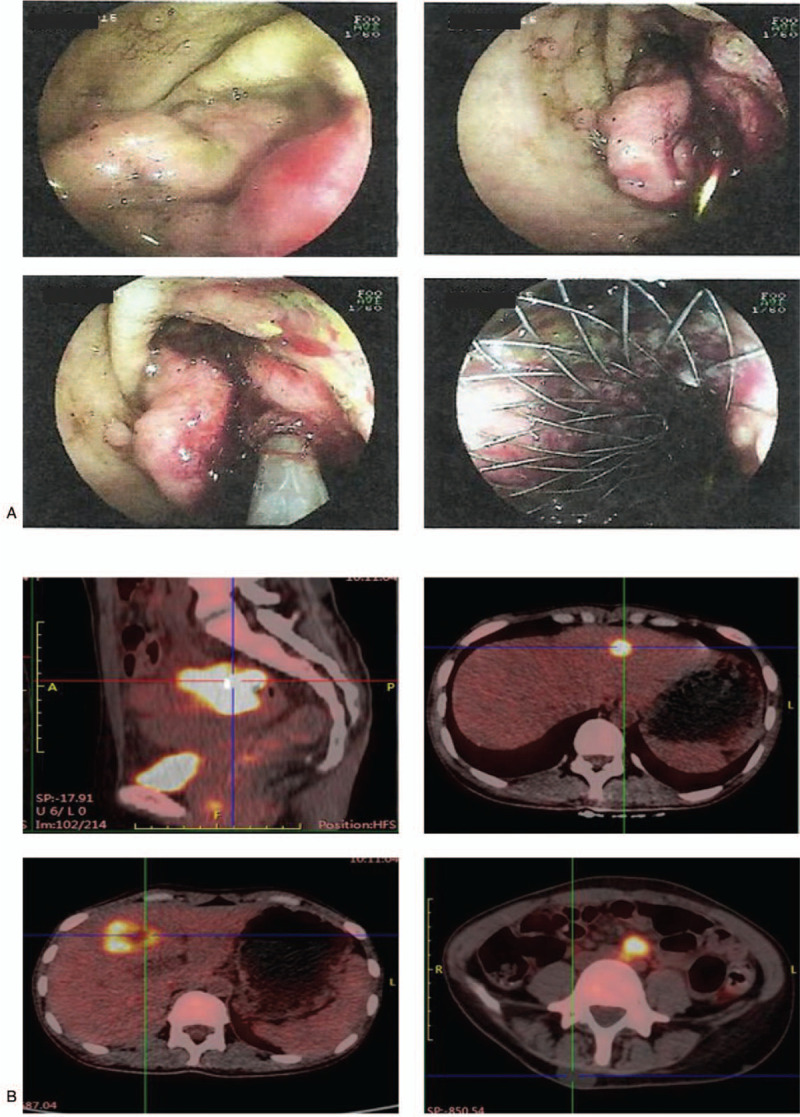
Gastroscopy revealed numerous sessile polyps of 0.2 to 0.5 cm extending from the gastric cardia to duodenum (A) and positron emission tomography-computed tomography (PET-CT) revealing high signaling in the recto-sigmoid junction, 2 high signals in the liver S3 and S4, and swelling retroperitoneal lymph nodes (B).

A 22-year-old female with GS was referred to our institution in April 2020. Due to poor finance support, she did not undergo any physical examination until she presented with severe weight loss, abdomen pain, and visible blood in the stool. On admission, the full blood count identified a hemoglobin level of 50 g/L. Intraoral clinical examination showed irregular teeth growth and panoramic radiographic examination revealed the presence of supernumerary teeth (Fig. 1A–C). General examination revealed multiple soft tissue swellings over the lower back and shoulder, which was suggestive as epidermoid cyst (Fig. 1D). Gastroscopy revealed numerous sessile polyps of 0.2 to 0.5 cm extending from the gastric cardia to duodenum, biopsy of which revealed adenomatous polyp (Fig. 2). Colonoscopy was performed per rectum identifying a malignant appearing lesion impending into the bowel at 15 cm above the anal verge, leading to construction of the bowel. Transverse loop colostomy was performed as an emergency to relieve the obstruction. Meanwhile, multiple sessile polyps were scattered around the rectum (Fig. 3A). The biopsy revealed adenocarcinoma. Positron emission tomography-computed tomography revealed high signaling in the recto-sigmoid junction, 2 high signals in the liver S3 and S4, and swelling retroperitoneal lymph nodes, indicating metastatic tumors with stage IV (Fig. 3B). Family history showed that 4 of the 5 brothers of her grandfather died of liver, lung, and gastric malignancy, but further details were not known.

First, the bowel obstruction was relieved by stent implantation. Chemotherapy regimen containing oxaliplatin and 5-Fu was administrated for 4 cycles, but the disease progressed. After multiple discipline discussion, tumor resection was tried, but failed due to extensive loco-regional nodal spread and peritoneum dissemination. After abdominal exploration, the patient was discharged from the hospital, but recovered very slowly. She refused to take further therapy and died of disease progression 12 weeks after discharge from hospital.

**Figure 4:**
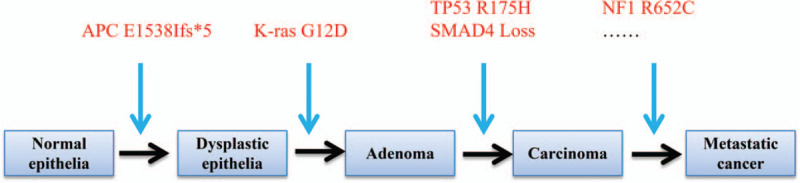
Accumulated acquired-mutations in key cancer-associated genes contributing early-onset and metastasis of tumors.

NGS sequencing searching for mutations from 365 genes and gene-fusion from 25 genes for the tumor tissues was performed by 3DMed Company. A frameshift variant in APC (E1538Ifs∗5) was identified in the tumor specimen and was determined to be a de novo germline mutation. This finding supports the diagnosis as GS. Additional somatic mutations in cancer-associated genes included mutations in K-ras (G12D), NF1 (R652C), TP53 (R175H), IRF2 (p.R82S), TCF7L2 (p.A418Tfs∗14), and SMAD4 (p.L43F) and a copy loss of SMAD4 (Fig. 4).

## 3 Discussion

The combination of multiple polyps in stomach, duodenum, and colon, together with epidermoid cyst, dental abnormalities signifies towards GS, which is further confirmed by *APC* gene defects. Although 2 potential targets, KRAS G12D and NF1 R652C, were found, the patient declined to receive targeted therapy of everolimus because of the patient's limited finances.

The majority of patients with FAP will develop colorectal carcinoma by the age of 40 to 50 years if the polyps in the colon are left untreated.^[5,10]^ In this case, colorectal carcinoma developed in adolescent, possibly due to accumulated acquired-mutations in multiple key cancer-associated genes. Specifically, the KRAS and TP53 mutations we identified are previously described as functionally acquired mutations that lead to sustained activation of the PI3K/Akt signaling pathway, leading to early onset of a variety of tumor types.^[11–14]^ Therefore, early detection and intervention seems to be essential for patients with GS. As for early detection, it is recommended that flexible proctosigmoidoscopy should be performed at the age of 10 to 12 years; repeat every 1 to 2 years until the age of 35; after the age of 35 years repeat every 3 years, while upper GI endoscopy should be performed every 1 to 3 year once when polyps are first identified.^[15]^ As for chemoprevention, nonsteroidal anti-inflammatory drugs can reduce the incidence of colorectal adenomas in patients with FAP and hereditary nonpolyposis colorectal cancer, indicating an effective alternative for the chemoprevention of malignant transformation of FAP (including GS) and hereditary nonpolyposis colorectal cancer.^[15–17]^

The clinical presentation of GS is variable. However, about 25% patients have no family history,^[18]^ which might delay the diagnosis, despite the presence of clues including dental abnormalities and extracolonic manifestations for a long time.^[19]^ This patient presented with multiple soft tissue swellings at the lower back at 5-year old and irregular teeth when she was a teenager. Unfortunately, she did not receive any appropriate evaluation and lost the chance of cure because of unawareness of this disease.

Surgical resection is supposed to the most effective way to treat this disease, other therapeutic options such as chemotherapy and radiation treatment didn’t produce satisfactory outcomes.^[20–23]^ However, the goal of R0 resection is not always possible because of the multiple polyps scattered in all the part of the gastrointestinal tract from the stomach to the rectum. Therefore, various therapeutic approaches have been proposed, including anti-inflammatory drugs, cytotoxic chemotherapy, radiotherapy, and tyrosine kinase inhibitors. To date, none have produced satisfactory results. In this case, we found 2 possible therapeutic targets, KRAS G12D for trametinib, NF1 R652C for trametinib, everolimus, and temsirolimus, in other tumors. However, the patient did not have the chance to try it because of the expensive price and rapid progression of the disease. Whether this kind of patients with GS harboring KRAS G12D or NF1 R652C will be benefit from targeted therapy should be further explored.

In conclusion, NGS assay is able to identify key molecular alteration for patients with FAP (including GS). However, to date, there are not too much therapeutic strategies for GS patients once the malignancies were formed. The best method is to detect the syndrome at early stage and prevent the malignant transformation of the polyps with drugs or surgery.

## Author contributions

**Resources:** Xin Li, Jiahua Xu, Jinzu Yang, Hongwei Li.

**Writing – original draft:** Xiaoqiang Gu, Xin Li.

**Writing – review & editing:** Jianxin Qian, Qing Wu.
